# Alteration of Tight Junction Gene Expression by Calciumand Vitamin D-Deficient Diet in the Duodenum of Calbindin-Null Mice

**DOI:** 10.3390/ijms141122997

**Published:** 2013-11-20

**Authors:** Inho Hwang, Hyun Yang, Hong-Seok Kang, Changhwan Ahn, Eui-Ju Hong, Beum-Soo An, Eui-Bae Jeung

**Affiliations:** 1 Laboratory of Veterinary Biochemistry and Molecular Biology, College of Veterinary Medicine, Chungbuk National University, Cheongju, Chungbuk 361-763, Korea; E-Mails: darkpower777@nate.com (I.H.); anpoong@naver.com (H.Y.); khskhs100479@naver.com (H.-S.K.); prac@naver.com (C.A.); euijuhong@hotmail.com (E.-J.H.); 2 Department of Biomaterials Science, College of National Resources & Life Science, Pusan National University, 1268-50 Samrangjin-ro, Samrangjin-eup, Miryang-si, Gyeongsangnam-do 627-706, Korea; E-Mail: anbs@pusan.ac.kr

**Keywords:** calbindin, transcellular pathway, paracellular pathway, calcium absorption, tight junction

## Abstract

Calcium absorption is regulated by both active (transcellular) and passive (paracellular) pathways. Although each pathway has been studied, correlations between the two pathways have not been well elucidated. In previous investigations, the critical transcellular proteins, calbindin-D_9k_ (CaBP-9k) and -D_28k_ (CaBP-28k), were shown to affect other transcellular pathways by buffering intracellular calcium concentrations. The rate of paracellular calcium transport in the duodenum is generally determined by the expression of tight junction genes. In the present study, the effect of dietary calcium and/or vitamin D supplementation on the expression of tight junction genes (*occludin*, *ZO-1* and *claudin 2*, *10b, 12* and *15*) in the duodenum of CaBP-9k- and/or -28k-deficient mice was examined. With a normal diet, the expression of most tight junction genes in the duodenum was significantly increased in CaBP-9k knockout (KO) mice compared to wild-type (WT) animals. With a calcium- and vitamin D-deficient diet, tight junction gene expression was significantly decreased in the duodenum of the CaBP-9k KO mice. These findings suggest that expression of paracellular tight junction genes is regulated by transcellular CaBP proteins, suggesting that active and passive calcium transport pathways may function cooperatively.

## 1. Introduction

Calcium is involved in many functions, such as intracellular signaling, blood clotting and muscle contraction, making this mineral essential for maintaining life. Therefore, a suitable amount of calcium intake and functional calcium (re)absorption mechanisms are important. Calcium transport mechanisms can be divided into active transcellular and passive paracellular pathways [1,2].

In transcellular pathways, transient receptor potential cation channel subfamily V members (TRPVs) 6, sodium-calcium exchanger (NCX) 1, plasma membrane calcium ATPase (PMCA) 1b, and calbindin-D9k/-D28k (CaBP-9k/28k) are involved in calcium transport. TRPV6 is a channel protein that facilitates the intake of luminal calcium ions into the cell. TRPV6 promotes calcium absorption in the duodenum [3–5]. NCX1 and PMCA1b are responsible for intracellular calcium ion excretion. NCX1 exchanges outer sodium ions for inner calcium ions [6,7]. PMCA1b facilitates the excretion of calcium ions using adenosine triphosphate (ATP) hydrolysis [8]. CaBP-9k and -28k help to buffer intracellular calcium concentrations in the duodenum and kidney, respectively [9–11].

In the paracellular pathways, tight junction genes influence calcium transport. Most tight junctions are apically positioned among intercellular junctions. Tight junctions are involved in several different processes, such as cell adhesion, intracellular signaling, protection from extracellular invasion and paracellular transport [12]. These junctions are composed of transmembrane proteins, cytoskeleton components and cytoplasmic plaques [13]. Among the various tight junction proteins, transmembrane proteins and cytoplasmic plaques are important for paracellular transport. Junction adhesion molecules (JAMs), occludin (OCLN) and claudin (CLDN) are representative transmembrane proteins [14]. These proteins close intercellular junctions and restrict the free movement of materials through the paracellular space. Therefore, non-selective and charge-selective ion transport are governed by the expression of transmembrane proteins [15]. Cytoplasmic plaques, such as zona occludens (ZO) proteins, include PSD-95/Dlg/ZO-1 (PDZ), which contain a binding domain for transmembrane proteins [16].

The CLDN family consists of tetraspan transmembrane proteins and includes 27 members. CLDNs have two extracellular loops (ECL): a long ECL1 and a short ECL2 [17]. Some of these proteins form charge-selective channels. The charge selectivity of each CLDN is determined by amino acids composing the ECL1 of each CLDN [18]. In the intestine, CLDN2, CLDN12 and CLDN15 are responsible for transporting calcium ions [19,20]. Other CLDNs, such as CLDN1 and CLDN5, have been shown to have clear sealing functions that may also affect calcium transport, because they influence general paracellular permeability [21,22].

OCLN is a tetraspan transmembrane protein. Unlike CLDNs, OCLN does not have charge selectivity, although it is critical for paracellular permeability [23,24]. The precise function of OCLN is still unclear. ZO-1 is a cytoplasmic plaque protein located in the border of the cellular plasma membrane. ZO-1 serves as a scaffold for transmembrane proteins by providing PDZ domains and also binds to cytoskeleton components, such as actin [16,25]. It has been reported that the phosphorylation levels of ZO-1 determine paracellular permeability [26].

Although both transcellular and paracellular transport systems have been actively studied, interactions between these systems are not well understood [27]. In the present study, it was hypothesized that transcellular and paracellular systems may be correlated. Therefore, insufficient transcellular pathway function due to an absence of CaBPs may induce the compensatory expression of both transcellular and paracellular tight junction genes. The expression of tight junction genes (OCLN, ZO-1 and CLDN) was evaluated in the duodenum of CaBP-9k/-28k single knockout (KO) or double KO (DKO) mice. Furthermore, the animals were fed calcium-deficient or calcium/vitamin D-deficient diets in order to further investigate the functions of tight junction genes in calcium absorption.

## 2. Results

### 2.1. Regulation of Serum Calcium Concentrations after the Consumption of Calcium-Deficient and Calcium/Vitamin D-Deficient Diets

Serum calcium concentrations according to genotype and diet were first examined. Calcium concentrations in the serum of all mouse strains were not significantly different, although that of the CaBP-28k KO mice was relatively low (Table 1). As expected, the serum calcium concentrations were significantly decreased when calcium-deficient or calcium/vitamin D diets were administrated. The calcium-deficient diets significantly decreased serum calcium concentrations in wild-type (WT), CaBP-9k KO and DKO mice, while the concentration was still in the normal physiological range (9.4–9.52 mg/dL) [28]. Calcium concentrations were decreased below normal ranges in WT, CaBP-28k KO and DKO mice fed the calcium/vitamin D-deficient diet. This reduction was the greatest in DKO mice. Interestingly, serum calcium concentrations of the calcium/vitamin D-deficient CaBP-9k KO animals were similar to those of CaBP-9k KO mice fed the calcium-deficient diet.

### 2.2. Expression of Tight Junction Genes in the Duodenum

**Figure 1:**
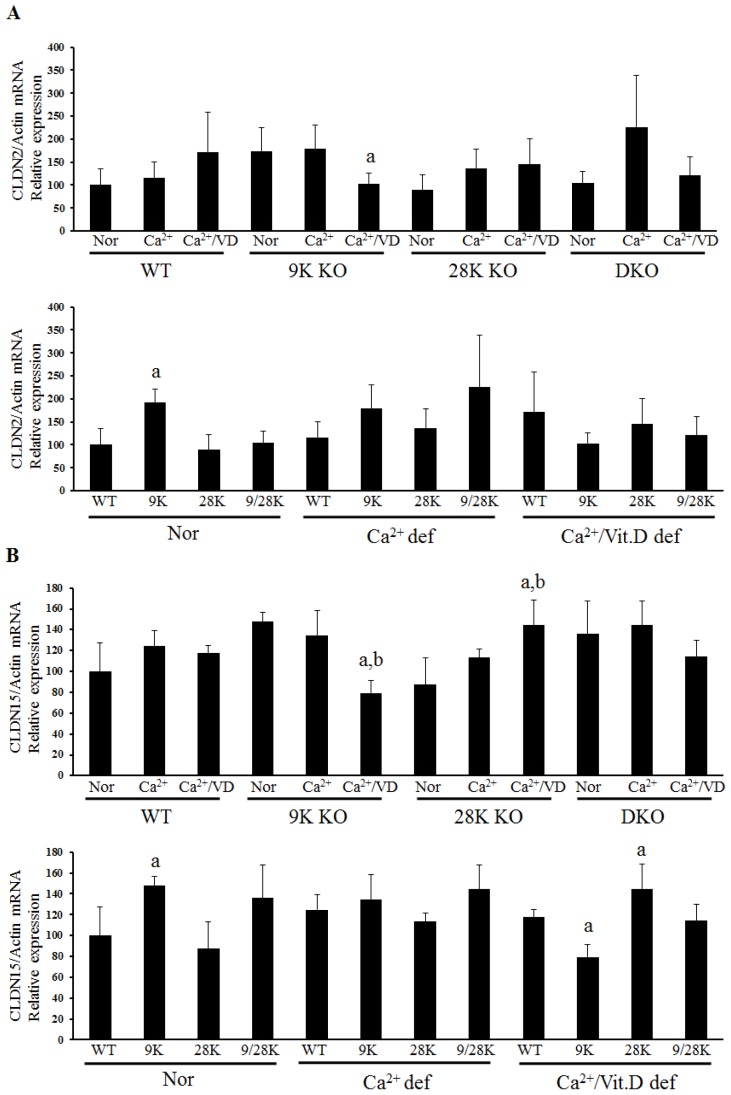
Tissue-specific tight junction genes expression in the duodenum of mice. *CLDN2* (**A**) and *CLDN15* (**B**) mRNA expression were analyzed by real-time PCR. The expressions of each gene were compared through the type of animal (**upper panel**) and diets (**lower panel**). Every result was normalized relative to β-actin. In upper panel of **(A)** and **(B)**, Nor, normal diet; Ca^2+^, calcium-deficient group; Ca^2+^/VD, calcium- and vitamin D-deficient diet; a, *p* < 0.05 (*vs.* normal diet) and b, *p* < 0.05 (*vs.* calcium deficient diet). In lower panel of **(A)** and **(B)**, WT, wild-type; def, deficient diet; 9k, CaBP-9k KO mice; 28k, CaBP-28k KO mice; 9k/28k, DKO mice; a, *p* < 0.05 (*vs.* wild-type mice).

The mRNA expression of tight junction genes in the duodenum was analyzed by real-time polymerase chain reaction (PCR) (Figure 1 and Table 2). Tight junction gene expression was highly regulated in CaBP-9k KO mice that were fed the calcium/vitamin D-deficient diet. The expression of *OCLN*, *ZO-1*, *CLDN2* and *CLDN15* was significantly decreased in CaBP-9k KO mice given the calcium/vitamin D-deficient diet compared to ones fed the normal diet (OCLN and ZO-1 data were not shown). Furthermore, compared with normal diet groups, *OCLN*, *CLDN2* and *CLDN15* expressions were significantly upregulated in CaBP-9k KO animals compared to their WT counterparts. *OCLN* expression was decreased in CaBP-9k KO mice that consumed the calcium/vitamin D-deficient diet compared to ones fed the normal diet. Calcium/vitamin D deficiency significantly reduced *ZO-1* mRNA expression in CaBP-9k KO mice compared to WT animals. *CLDN2* (Figure 1A) showed expression pattern similar to those of *OCLN. CLDN10b* expression was upregulated in calcium/vitamin D-deficient WT mice, as well as calcium-deficient CaBP-9k KO mice. CaBP-28k KO and DKO mice fed the normal diet had higher levels of *CLDN10b* expression than WT animals. *CLDN10b* was highly expressed only in CaBP-9k KO mice fed the calcium-deficient diet compared to the WT mice. *CLDN 12* mRNA expression was upregulated in the calcium/vitamin D-deficient CaBP-9k KO mice relative to the WT animals. *CLDN15* levels were also significantly decreased in CaBP-9k KO mice fed the calcium/vitamin D-deficient diet compared to ones given the normal diet (Figure 1B). Among the CaBP-28k KO mice, *CLDN15* expression was upregulated in the calcium/vitamin D-deficient group compared to the normal diet group. Among calcium/vitamin D-deficient animals, *CLDN15* mRNA expression was significantly lower in CaBP-9k KO mice than WT mice, while it was significantly higher in CaBP-28k KO mice compared to the WT animals. Expression patterns of the tested genes in the duodenum were represented in Table 2.

### 2.3. Regulation of Duodenal Tight Junction Protein Expression

**Figure 2:**
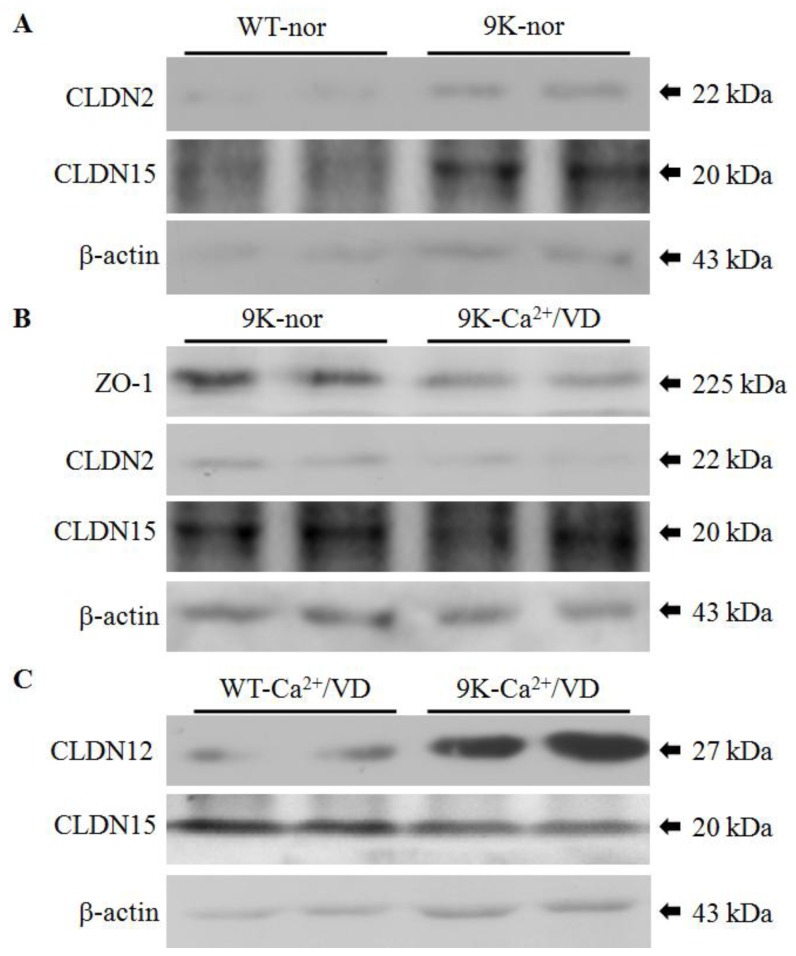
Regulation of tight junction protein expression in the duodenum. ZO-1, CLDN2, 12 and 15 protein expression was measured by Western blotting. The levels of CLDN2, and CLDN15 proteins in WT and CaBP-9k KO mice were compared (**A**); ZO-1, CLDN2 and CLDN15 protein expression levels were compared between normal and calcium/vitamin D-deficient CaBP-9k KO mice (**B**); In calcium- and vitamin D-deficient WT and CaBP-9k KO mice, CLDN12 and CLDN15 protein expression levels were compared (**C**); β-actin was used as an internal control for each experiment. 9k, CaBP-9k KO mice; nor, normal diet; Ca^2+^/VD, calcium/vitamin D-deficient diet.

The expression and function of CaBP-9k and -28k are tissue-specific. CaBP-9k is predominantly expressed and primarily functions in the duodenum, whereas CaBP-28k is kidney-specific [29]. To further evaluate the regulation of tight junction gene expression, we analyzed the expression of tight junction proteins in the intestine of CaBP-9k KO mice. The tight junction proteins were selected based on mRNA results in this study. The levels of ZO-1 and CLDN2, 12 and 15 proteins were measured by Western blotting (Figure 2). With the normal diet, CLDN2 and CLDN15 protein expressions were upregulated in CaBP-9k KO mice compared to the WT animals (Figure 2A), and ZO-1, CLDN2 and CLDN15 protein expressions were downregulated in the calcium/vitamin D-deficient groups (Figure 2B). ZO-1 and CLDN12 protein levels were higher in WT mice fed the calcium/vitamin D-deficient diet compared to the CaBP-9k KO mice, while CLDN15 protein expression was increased (Figure 2C). These patterns of protein expression were consistent with ones observed for mRNA.

### 2.4. Localization of Duodenal Tight Junction Proteins

**Figure 3:**
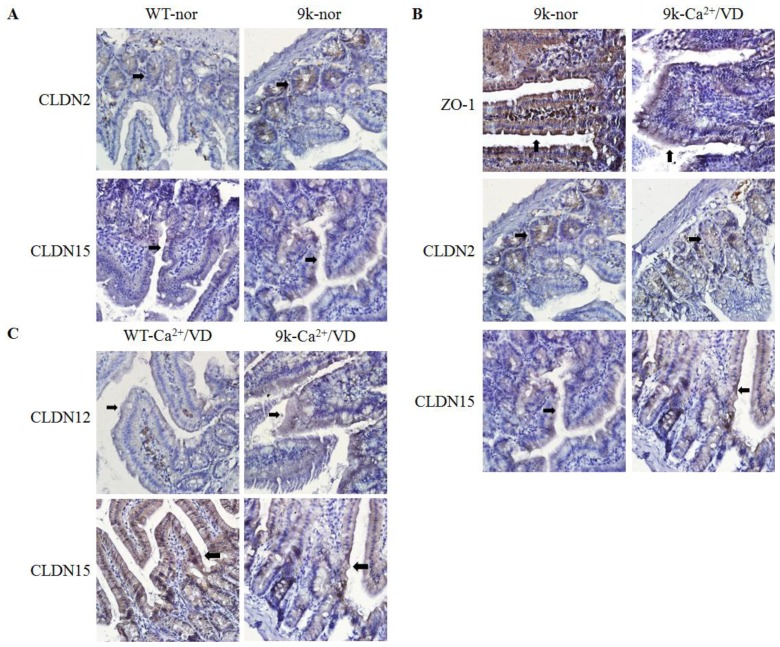
Localization of tight junction proteins in the duodenum. Differential localization of ZO-1, CLDN2, CLDN12 and CLDN15 was observed with immunohistochemistry. CLDN2 and CLDN15 localization was compared in the duodenum of WT and CaBP-9k KO mice fed the normal diet (**A**); The dietary effect of calcium/vitamin D deficiency on ZO-1, CLDN2 and CLDN15 localization in the duodenum of CaBP-9k KO mice was examined (**B**); Differential CLDN12 and CLDN15 localization in the duodenum of calcium/vitamin D-deficient WT and CaBP-9k KO mice was evaluated (**C**). Each slide was viewed at 400× magnification. Black arrows indicate the immuno-positive signals.

The localization of ZO-1, CLDN2, CLDN12 and CLDN15 proteins was also examined (Figure 3). All tight junction proteins were found in the duodenal villi epithelium. Strong immunohistochemical signals were observed in CaBP-9k KO mice fed the normal diet compared to the WT control, although the localization patterns were not altered (Figure 3A). In the CaBP-9k KO mice, immuno-positive signals were weaker in the duodenal villi of calcium/vitamin D-deficient animals than mice fed the normal diet (Figure 3B). With the calcium/vitamin D-deficient diet, CLDN15 signals were weaker in CaBP-9k KO mice than the WT animals, but CLDN12-specific signals were stronger in the CaBP-9k KO mice compared to the WT mice (Figure 3C). Results of the immunostaining experiment concurred with the western blot results (Figure 2).

## 3. Experimental Section

### 3.1. Animals

Age- and gender-matched (3-week old males) wild-type (WT; C57BL/6), CaBP-9k KO, CaBP-28k KO and CaBP-9k/-28k DKO mice were used in this study. The WT animals were obtained from KOATECH (Pyeongtaek-si, Gyeonggi-do, Korea). Mice lacking CaBP-9k and/or -28k expression were generated, and the genotypes of the offspring were determined as previously described [30]. Briefly, CaBP-9k KO, CaBP-28k KO and DKO mice were produced by breeding the corresponding heterozygous mice. Genotypes of the mice were determined by genomic PCR analysis using tail tissue DNA. A total of 60 animals were divided into 12 groups (*n* = five per group), according to genotype and diet-type.

### 3.2. Experimental Treatments

To investigate the dietary effects of calcium and vitamin D, the mice were fed a normal diet (DYET #113295, AIN-76A purified rodent diet containing 0.8% phosphorus and 1.1% calcium; Dyets Inc., Bethlehem, PA, USA), a calcium-deficient diet (DYET #113294, AIN-76A purified rodent diet with 1% phosphorous and 0.02% calcium; Dyets Inc.) or a calcium-/vitamin D-deficient diet (D10373A, AIN-76A-based diet containing 0.8% strontium, 0.02% calcium and 0.35% phosphorus; Research Diets, Inc., Brunswick, NJ, USA). All animals were fed the normal or experimental diets for 4 weeks (when the mice were 3 to 7 weeks old). All the mice were then euthanized with ether, and tissue samples from the duodenum were collected. All animal experimental procedures were approved by the Ethics Committee of Chungbuk National University in the Republic of Korea.

### 3.3. Serum Calcium Concentration Analysis

Blood samples were collected from the abdominal vein of each mouse, transferred to serum separator tubes (BD Caribe, Ltd., Franklin Lakes, NJ, USA), and centrifuged at 400× *g* for 15 min. The serum Ca^2+^ concentrations were measured using an auto analyzer (Modular Analytics, Roche, Mannheim, Germany).

### 3.4. Quantitative Real-Time PCR

Total RNA was extracted using TRIzol reagent (Ambion, Austin, TX, USA), according to the manufacturer’s instructions. The total RNA concentration was measured at 260 nm with an Epoch micro-volume spectrophotometer (BioTeK, Winooski, VT, USA). First strand complement DNA (cDNA) was synthesized by reverse transcription from 1 μg of total RNA using Moloney murine leukemia virus (MMLV) reverse transcriptase (Invitrogen Co., Carlsbad, CA, USA) and random primers (9-mers; TaKaRa Bio Inc., Otsu, Shiga, Japan). Reverse transcription (RT) PCR was performed with a 7300 Real-Time PCR system (Applied Biosystems, Foster City, CA, USA), according to the manufacturer’s instruction. β-actin was used as an internal control for normalization and the relative gene expression levels were quantified using RQ software (Applied Biosystems).

Quantitative real-time PCR was performed for reactions containing 1 μL of cDNA template with 10 pmol of primers specific for tight junction genes and 10 μL of 2 × SYBR Premix ExTaq (TaKaRa Bio Inc.). The primer sequences are listed in Table 3. PCR was carried out for 40 cycles of denaturation at 95 °C for 15 s, annealing at 62 °C for 15 s and extension at 72 °C for 30 s. At the end of the extension phase of each cycle, fluorescence intensity was measured, and the threshold level was manually set for each sample. The threshold cycle (CT) was defined as the cycle when sample fluorescence reached the threshold level.

### 3.5. Western Blot Analysis

Proteins were extracted with Pro-Prep (iNtRON Biotechnology, Gyeoggi-Do, Korea) and homogenized. The protein samples were centrifuged at 14,000 rpm, separated in 7.5%–12.5% SDS-PAGE gels (40 μg per lane) and transferred to nitrocellulose membranes (Millipore, Bedford, MA, USA). The membranes were blocked with 5% skim milk in Tris-buffered saline with 0.5% Tween-20 (TBS-T) for 2 h at room temperature and then incubated overnight (O/N) at 4 °C with the following primary antibodies: rabbit anti-ZO-1 (1:1000; Invitrogen Co.), anti-CLDN12 (1:1000; Invitrogen Co.), anti-β-actin (1:1000; Santa Cruz Biotechnology, Dallas, TX, USA), anti-CLDN15 (1:1000; Invitrogen Co.) and mouse anti-CLDN2 (1:1000; Invitrogen Co.). Next, the membranes were washed with TBS-T for 1 h at room temperature and incubated with anti-rabbit and anti-mouse horseradish peroxidase-conjugated secondary antibodies (1:3000; Santa Cruz Inc.) for 2 h at room temperature. After subsequently washing the membranes with TBS-T, antibody binding was detected with an enhanced chemiluminescence reagent (Amersham Biosciences, Little Chalfont, UK) and detected by ChemiDoc equipment GenGnome 5 (Syngene, Cambridge, UK). To ensure signal specificity, the membranes were incubated with the secondary antibody alone.

### 3.6. Immunohistochemistry

Tissue-specific localization of tight junction proteins was examined using immunohistochemistry. Samples of the duodenum were embedded in paraffin, cut into 5-μm sections, deparaffinized with xylene and hydrated in descending graded ethanol solutions. The sections were then mounted onto glass slides (Matunami, Ishikawa, Japan) coated with amino silane (APS). Endogenous peroxidase activity was blocked by incubating the slides with 3% hydrogen peroxidase in phosphate buffered saline (PBS) for 30 min at room temperature. To prevent non-specific reactions, the sections were incubated with 10% goat serum (Vector Laboratories, Burlingame, CA, USA) in PBS for 1 h at room temperature. After washing with TBS-T, the sections were incubated overnight at room temperature with the same primary antibodies used for Western blotting (rabbit anti-ZO-1, -CLDN12 and -CLDN15; and mouse anti-CLDN2) diluted 1:250 with 5% bovine serum albumin (BSA). The slides were washed with TBS-T before being incubated with biotinylated secondary antibodies (1:500, rabbit or mouse IgG; Vector Laboratories, Inc.) for 1 h at 37 °C and then ABC Elite solution (Vector Laboratories, Inc.) for 30 min at 37 °C. Diaminobenzidine (Sigma, St. Louis, MO, USA) was used as a chromogen. The sections were counterstained with hematoxylin and mounted in Cytoseal* 60 (Richard-Allan Scientific Co., Kalamazoo, MI, USA).

### 3.7. Data Analysis

Data were analyzed with a nonparametric one-way analysis of variance (ANOVA), followed by Tukey’s test for multiple comparisons. All statistical analyses were performed using SPSS for Windows (SPSS, Chicago, IL, USA). *p*-values < 0.05 were considered statistically significant.

## 4. Conclusions

Calcium has many essential physiological roles, but is also toxic at excessively high concentrations. Therefore, adequate calcium absorption and transport are important for living organisms. In duodenum, calcium is transported from the luminal space to blood through active transcellular and passive paracellular pathways [1,2]. Mechanisms governing these pathways have been extensively studied. However, interaction between the two pathways has not been investigated in great detail. In the present study, it was assumed that insufficient transcellular pathway functioning may induce compensatory paracellular pathway activity in the duodenum. Therefore, the regulation of paracellular tight junction gene expression in the duodenum of CaBP-9k or -28k KO and DKO mice was evaluated. Furthermore, the animals were fed calcium- or calcium/vitamin D-deficient diets to study the effects of dietary calcium on tight junction gene expression.

The concentrations of calcium in the body can be estimated by measuring serum calcium concentrations. In the present study, no obvious changes in serum calcium were observed after consumption of the calcium-deficient diet. In contrast, the calcium/vitamin D-deficient diet decreased serum calcium concentrations below the normal physiological range in each strain of mice, except for the CaBP-9k KO animals. Regardless of diet, calcium concentrations were higher in the CaBP-9k KO mice. In the DKO mice, the calcium/vitamin D-deficient diet dramatically decreased serum calcium concentrations compared to the other strains of animals. Therefore, we assumed that tight junction gene expression may be more tightly regulated in the CaBP-9k KO mice than the CaBP-28k KO or DKO mice to maintain calcium concentrations in the blood.

The expression patterns of tight junction genes were similar in the intestine. We measured the expression of *OCLN*, *ZO-1*, *CLDN2*, *CLDN10b*, *CLDN12* and *CLDN15* genes in the duodenum. *OCLN* is a transmembrane protein that is apparently associated with paracellular permeability [21–24]. *CLDN2*, *CLDN12* and *CLDN15* are responsible for calcium absorption in the intestine, and *CLDN10b* has been reported to form cation-selective channels [19,20,31,32].

In the duodenum, CaBP-9k is a major factor in the transcellular calcium transport pathway [9]. The expression of tight junction genes in the duodenum was generally upregulated with the normal diet in CaBP-9k KO mice compared to WT mice and downregulated in animals fed the calcium/vitamin D-deficient diet compared to ones that consumed the normal diet. These results suggest that there was compensatory overexpression of the tight junction genes due to insufficient transcellular calcium absorption resulting from the ablation of CaBP-9k expression. It is also possible that CaBP-9k directly influences the tight junction system. In the CaBP-9k KO mice, calcium/vitamin D-deficiency led to decreased expression of most tight junction genes that were evaluated. Decreased *CLDN2* and *CLDN15* expressions in the intestine indicated that a lack of calcium absorption due to calcium and/or vitamin D deficiencies leads to reduced tight junction gene expression, limiting calcium outflow, given that paracellular transport is bidirectional.

Vitamin D is thought to be critical for the regulation of tight junction, because the levels of tight junction proteins, such as CLDN, OCLN and ZO-1, were affected by vitamin D deprivation. Furthermore, CaBP-9k is known to be a vitamin D-dependent protein [33].

Previously we examined the effect of CaBP-9k on the regulation of the vitamin D receptor (VDR) [34,35]. In this study, VDR was not regulated significantly in CaBP-9k KO mice, while the parathyroid hormone receptor (PTHR) mRNA was significantly reduced in CaBP-9k KO compared to WT mice. Interestingly, the *PTHR* gene was oppositely upregulated in CaBP-9k KO mice when fed with calcium- and vitamin D-deficient diets. These results led us to test the CaBP regulation of paracellular calcium transport proteins with and/or without calcium and vitamin D supplementations. Although the main genotype of CaBP in the intestine is CaBP-9k, we also tested CaBP-28k KO mice to test the possible relationship of CaBP-28k with CaBP-9k and paracellular calcium transporters.

In this study, *CLDN2* and *CLDN15* transcripts were upregulated in the absence of the CaBP-9k gene, suggesting that these CLDNs are correlated with CaBP-9k. It is possible that narrowed calcium absorption pathway due to the lack of the CaBP-9k-induced transcellular pathway stimulates alternative paracellular calcium absorption pathways via CLDN2 and CLDN15 proteins. These compensatory pathways were inhibited when the mice were fed with calcium/vitamin D-deficient diets, meaning that the compensatory pathways were allowed only in the presence of vitamin D, the pivotal regulator of calcium absorption in the intestine. Further studies are required to clarify the network of transcellular and paracellular pathways for calcium absorption.

In summary, the results of the current study demonstrated that ablation of CaBP-9k affects the expression of paracellular tight junction genes. Transcellular CaBP-9k, but not CaBP-28k, is associated with the compensatory expression of paracellular tight junction genes in the duodenum. Dietary calcium and vitamin D may also be necessary for this compensation. Taken together, our findings indicated that calcium (re)absorption is regulated by the network of transcellular and paracellular pathways.

## Figures and Tables

**Table 1:** Regulation of serum calcium concentrations (mg/dL) in CaBP knockout (KO) mice given a calcium-deficient or calcium/vitamin D-deficient diet. DKO, double KO.

Diet	WT	CaBP-9K KO	CaBP-28K KO	DKO
Normal	9.6 ± 0.15	9.8 ± 0.12	9.2 ± 0.10	9.6 ± 0.10
Ca^2+^ def	9.4 ± 0.06 *	9.4 ± 0.06 *	9.2 ± 0.06	9.0 ± 0.10 *
Ca^2+^/VD def	8.3 ± 0.15 **	9.4 ± 0.12 *	8.2 ± 0.06 **	7.5 ± 0.06 **

Notes:

* significantly lower than the normal diet group (*p* < 0.05);

** significantly lower than the calcium-deficient group (*p* < 0.05);

Ca^2+^ def, calcium-deficient diet; Ca^2+^/VD def, calcium/vitamin D-deficient diet; VD, vitamin D.

**Table 2:** Tight junction gene regulation in the duodenum.

Diet	WT	CaBP-9K KO	CaBP-28K KO	DKO
Normal	-	OCLN↑, CLDN2↑, CLDN15↑	CLDN10b↑	CLDN10b↑
Ca^2+^ def	-	CLDN10b↑	-	-
Ca^2+^/VD def	-	OCLN↓, ZO-1↓, CLDN12↑ CLDN15↓	CLDN15↑	-

Notes:

↑ significantly higher than same conditioned WT mice (*p* < 0.05);

↓ significantly lower than same conditioned WT mice (*p* < 0.05;

-, no comparison; Ca^2+^ def, calcium-deficient diet; Ca^2+^/VD def, calcium/vitamin D-deficient diet.

**Table 3:** List of primers used for polymerase chain reaction (PCR) in this study.

Gene	Forward	Reverse
***β-actin***	5′-ACAGGCATTGTGATGGACTC-3′	5′-ATTTCCCTCTCAGCTGTGGT-3′
***OCLN***	5′-ACTGGGTCAGGGAATATCCA-3′	5′-TCAGCAGCAGCCATGTACTC-3′
***ZO-1***	5′-ACTCCCACTTCCCCAAAAAC-3′	5′-CCACAGCTGAAGGACTCACA-3′
***CLDN2***	5′-TGGTTCCTGACAGCATGAAA-3′	5′-CTTTGGGCTGTTGAGCAGAT-3′
***CLDN10b***	5′-TCGCCTTCGTAGTCTCCATC-3′	5′-TCTCCTTCTCCGCCTTGATAC-3′
***CLDN12***	5′-AGGAAGTTTGAGCCGGTCTT-3′	5′-CGTGATGAATAGGGCTGTGA-3′
***CLDN15***	5′-GCCTCTTTCTAGGCATGGTG-3′	5′-TCCAGCATACAGTGGGTTGA-3′
